# Caveolin-1 Expression Level in Cancer Associated Fibroblasts Predicts Outcome in Gastric Cancer

**DOI:** 10.1371/journal.pone.0059102

**Published:** 2013-03-19

**Authors:** Xianda Zhao, Yuyu He, Jun Gao, Lifang Fan, Zonghuan Li, Guifang Yang, Honglei Chen

**Affiliations:** 1 Department of Pathology, School of Basic Medical Science, Wuhan University, Wuhan, P. R. China; 2 Department of Molecular Pathology, Wuhan Nano Tumor Diagnosis Engineering Research Center, Wuhan, P. R. China; 3 Department of Pathology, Zhongnan Hospital of Wuhan University, Wuhan, P. R. China; 4 Department of Biochemistry, Rush University Medical Center, Chicago, Illinois, United States of America; Vanderbilt University Medical Center, United States of America

## Abstract

**Aims:**

Altered expression of epithelial or stromal caveolin-1 (Cav-1) is observed in various types of human cancers. However, the clinical significance of Cav-1 expression in gastric cancer (GC) remains largely unknown. The present study aims to explore the clinicopathological significance and prognostic value of both tumor cells and cancer associated fibroblasts (CAFs) Cav-1 in GC.

**Methods and Results:**

Quantum dots immunofluorescence histochemistry was performed to examine the expression of Cav-1 in 20 cases of gastritis without intestinal metaplasia (IM), 20 cases of gastritis with IM and 286 cases of GC. Positive rates of epithelial Cav-1 in gastritis without IM, gastritis with IM and GC showed a decreasing trend (*P* = 0.012). Low expression of Cav-1 in CAFs but not in tumor cells was an independent predictor of poor prognosis in GC patients (*P* = 0.034 and 0.005 respectively in disease free survival and overall survival). Cav-1 level in tumor cells and CAFs showed no significant correlation with classic clinicopathological features.

**Conclusions:**

Loss of epithelial Cav-1 may promote malignant progression and low CAFs Cav-1 level herald worse outcome of GC patient, suggesting CAFs Cav-1 may be a candidate therapeutic target and a useful prognostic marker of GC.

## Introduction

Like normal tissues, tumors are composed of two discrete but interactive compartments, the parenchyma and the stroma. In tumors, the tumor cells themselves are parenchyma, whereas the stroma includes a mixture of extracellular matrix (ECM) elements and non-malignant cells, such as cancer associated fibroblasts (CAFs), vascular endothelial cells and immune and inflammatory cells [1], [2], [3]. In recent years, the profound influences of tumor stroma on the growth and metastasis in various types of tumors have been widely elucidated [2], [3]. Tumor cells can trigger the deposition of a reactive stroma containing activated CAFs, immune and inflammatory cells, ECM elements that may favor invasion and metastasis of cancers [2], [3]. Besides, although definite roles of proteins in molecular cross talk between tumor and stromal cell remain unclear, altered expression of stromal proteins have been manifested as novel biomarkers in various types of human cancers, including breast [4], [5], [6], [7], prostate [8], nasopharynx [9] and basal cell cancer [10].

The caveolin gene family has three members: *CAV1*, *CAV2*, and *CAV3*, coding for the proteins caveolin-1 (Cav-1), caveolin-2 and caveolin-3, respectively [11]. *CAV1* is located on chromosome 7 (locus 7q31.1) and contains three exons (35, 165 and 324 bp) and two introns (1.5 and 32 kb) [11]. Cav-1 constitutes the major structural component of caveolae, which are flask-shaped vesicular invaginations of plasma membrane [12], [13]. Various receptors and signaling molecules are localized in the caveolae and are negatively regulated by Cav-1 through its scaffolding domain. Besides, Cav-1 interacts directly with the bilayer of cholesterol and sphingolipids within caveolin, therefore, influencing the lipid homeostasis and transport [12], [13]. In cancers, it is increasingly clear that Cav-1 is implicated in regulating multiple cancer-associated processes, ranging from cellular transformation, tumor growth, invasion and metastasis, to multidrug resistance and angiogenesis [14]. Cav-1 shows a compartment-dependent role on tumors. In the epithelial compartment, Cav-1 impacts both positively and negatively on tumor development. In tumor stroma, especially the CAFs, low expression of Cav-1 protein predicts adverse outcome in breast and prostate cancer [4], [5], [6],[8],[12],[15]. Nevertheless, clinical values of Cav-1 in GC remain not entirely clear. Based on prior researches, we hypothesis that roles of Cav-1 in epithelial and stroma may be different, roles of epithelial Cav-1 is uncertain but low expression of Cav-1 in CAFs may promotes GC progression and correlates with adverse outcome of GC patients.

To clarify the relationship between Cav-1 and GC progression or suppression, we have analyzed specifically both stromal and epithelial cell expression of Cav-1 in tissues sections, via the advanced quantum dots (QDs)-based immunofluorescence histochemistry (QDs-IHC) that had been developed in our previous studies and which have been widely accredited and used in laboratories [16], [17], [18], [19], [20], [21], [22]. Fluorescent semiconductor nanocrystal QDs are a novel class of multifunctional inorganic fluorophores that have many benefiting properties, such as narrow emission band peaks, wavelength of their fluorescence depends strongly on their sizes and different QD colors can be simultaneously excited by a single light source with minimal spectral overlapping [16], [17], [18], [19], [20], [21], [22]. These properties make QDs extremely useful for multiplexed molecular immunofluorescent imaging [21], [23]. The advanced multiple targets labeling technology of QDs-IHC enabled a precise analysis of Cav-1 expression in CAFs, by simultaneous detection of alpha-smooth muscle actin (α-SMA), which is a marker of CAFs and Cav-1 protein.

## Materials and Methods

### Patients and Follow-up

Because the time was limited and some patients were out of the hospital, so it is difficult to obtain written consent. We called to each patient, explained our study was used for academic exchanges only, and was not harmful to their health, and not contained their private information. When we called, a notary public was present, and we received the mobile phone short message which we required patients who consent the study to send to us. If the patient has dead, we got consent from his or her legal representative. Finally, we obtained verbal consent from all the 300 patients. A total of 340 formalin-fixed, paraffin-embedded tissues were obtained from patients diagnosed in the period from July 2005 to February 2012, including 300 GCs, 20 gastritis without intestinal metaplasia (IM) tissues and 20 gastritis with IM tissues. The gastritis without IM and with IM samples were derived from adenocarcinoma paracancerous tissues. Serial section confirmed no tumor tissue was found in these samples. Specimens were collected from the archives of the Department of Pathology, Zhongnan Hospital of Wuhan University (Hubei, P.R. China). Histological diagnosis and grades of differentiation were determined in accordance with the World Health Organization (WHO) criteria for GC. All of the GC samples were classified based on the UICC TNM classification (2009). Two board-certified pathologists (Yang GF and Fan LF) reconfirmed the histopathologic features of these samples. 326 samples were successfully preserved for further analysis; meanwhile, 14 GC samples were lost from the slide during immunostaining. Clinicopathological factors of GC patients were listed in Table 1. For 247 GC patients there was sufficient tissue for analysis of tumor cells and cancer associated fibroblastic Cav-1 immunostaining. All these 300 patients were treated with radical resection or cytoreductive surgery of GC, prior to administration of chemotherapy or radiotherapy. This study was approved by the Institute Research Medical Ethics Committee of School of Basic Medical Science, Wuhan University.

**Table 1:** Patient characteristics (N = 286).

Characteristic	Sub-characteristic	Value
Age(year)		57(24–92)
Gender	Male	205
	Female	81
UICC stage^#^	Ia/Ib	8/51
	II	83
	IIIa/IIIb	105/34
	IV	5
Pathologic type	AC	237
	Mucinous AC	32
	UC	17
Grade of AC	I	8
	II	70
	III	159
Lymph node status^#^	No	113
	Yes	173
HER 2 statues	Positive	34
	Negative	145
	Unknown	107
Lauren classification	Intestinal-type	137
	Diffuse-type	123
	Mixed-type	26

TNM classification of malignant tumors (7th edition).

AC, Adenocarcinoma; UC, Undifferentiated carcinoma.

Follow-up began on the date of surgery and ended in August 2012. Among the remaining 286 GC tissues from patients, 257 enrolled in our follow-up cohort and 116 patients who successfully reached a five year follow-up or died because of GC were included in the survival analysis. Patients, who did not reach five year follow-up and died of other diseases or due to unexpected events, were excluded from the survival study. The median follow-up of the 116 patients was 62 (range: 1–85) months. Overall survival was defined as the interval from the date of surgery to death. Disease free survival was defined as the interval from the date of surgery to recurrence. Diagnosis of recurrence was based on the following criteria: local recurrence found by endoscopic biopsy or with relaparotomy; metastasis on radiological, ultrasonic or cytological examination.

### Tissue Microarray Construction

Two sets of tissue microarrays (TMAs) were constructed. Each set contained 5 TMAs that were mounted with 340 specimens. The most representative tumor areas were defined on hematoxylin- and eosin-stained sections and marked on the slide. The way we constructed the TMAs and the microarray system instrument were the same with our previous study [24], [25]. As previously studies did not report intratumoral heterogeneity of Cav-1 expression in GC and our feasibility experiment indicated that one core per tumor on TMA and large sections showed a good consistency [26], [27], [28], therefore, in testing experiment, one core was sampled in each case. Briefly, one core with a diameter of 1.5 mm was taken from each sample and inserted into empty “recipient” paraffin blocks; these blocks were cut into sections (4 µm thick) and used for QDs-IHC analysis.

### QDs-based Immunofluorescence Histochemistry

Cav-1 immunoreactivity was detected in one set of TMAs. The antibodies, QDs conjugated streptavidin probes (QDs-SA) with 605 nm emission wavelength and related reagents for Cav-1 detection were the same as before [16] with the following major procedures: deparaffinizing → antigen retrieval → blocking → primary antibody for Cav-1(Cell Signaling Technology, Beverly, MA) → washing and blocking → biotinylated IgG →washing and blocking →605 nm-QDs-SA →washing →mounting and observation. The signal obtained from the labeling cells was detected via using Olympus BX51 fluorescence microscopy (CCD DP72). The QDs signal was target specific, red, and photostable. Autofluorescence of tissue-background was green. Since Cav-1 normally localizes in endothelial cells of blood vessels [27], [29] that examined in every TMA, it can be served as internal positive and quality control in QDs-IHC. TBS instead of Cav-1 primary antibody served as negative control.

### QDs-based Double Immunofluorescent Labeling

We used one set of TMAs for Cav-1/α-SMA colocalization, detected by QDs-based double immunofluorescent labeling technology according to the manufacturer’s instructions (Wuhan Jiayuan Quantum Dots Co., Ltd.). All dilution steps (antibodies and QDs) were performed in TBS containing 2% bovine serum albumin (BSA, Sigma, St. Louis, MO, USA). Antigen retrieval was performed in citric acid (10 mM, pH 6.0) at 95°C for 10 min, followed by cooling for 30 min. TMAs were first incubated in 2% BSA buffer at 37°C for 30 min, and then at 4°C overnight in rabbit anti-Cav-1 polyclonal antibody and mouse anti-α-SMA monoclonal antibody (1∶300 dilution, Abcam, Cambridge, MA) in order to antibody bindings. Then TMAs were washed three times with TBS-T (0.5% Tween in TBS) for 5 min each time, and incubated in biotinylated goat anti-mouse IgG (1∶400 dilution, Jackson ImmunoResearch, West Grove, PA) at 37°C for 30 min. For QDs conjugation, the antibody-binding TMAs were incubated in 2% BSA buffer at 37°C for 10 min, incubated in QDs (525 nm) conjugated to streptavidin (1∶200, Wuhan Jiayuan Quantum Dots Co., Ltd.) and QDs (605 nm) conjugated to goat anti-rabbit IgG (1∶100, Wuhan Jiayuan Quantum Dots Co., Ltd.) for 50 min, rinsed three times with TBS-T for 5 min each. Finally, TMAs were sealed with 90% glycerin (Sigma). The immunofluorescent signal was observed by Caliper’s multispectral microscopy imaging systems (Caliper Life Sciences). Because the immunofluorescent signal of single labeling and double labeling were snapped by CCD DP72 and multispectral microscopy imaging systems respectively, we calibrated results of the two instruments to ensure comparability of these experiments. Positive signal of α-SMA was green, and Cav-1 was red. Cav-1 expression in endothelial cells of blood vessels [27], [29], α-SMA expression in blood vessels and known positive tissue are regarded as the positive controls. TBS instead of two primary antibodies served as negative controls, only showing autofluorescence signal.

### Scoring of the Immunofluorescent Results

Results were evaluated by two pathologists (Yang GF and Fan LF) simultaneously in same displayer, who are independent and blinded to the clinical features of the study. The scores of the two pathologists were compared and any discrepant scores were reassessed by reevaluation of the stained tissue specimen by the two pathologists to achieve a consensus score. The methodology for scoring Cav-1 staining of tumor cells and CAFs were the same. Scores were determined by combining the proportion of positively stained cells and the intensity of staining. The sections were initially scanned at low power. Then cells from five representative fields of each specimen at high magnification (200×) were counted for Cav-1-stained tumor cells or CAFs. The area of positive (AP) are graded the cells as follows: 0 (no positive area or positive area <5%), 1 (positive area 5–25%), 2 (positive area 26–50%), 3 (positive area 51–75%) and 4(positive area >75%). Cav-1 intensity of staining (IS) by the numerical value is resulted in scores: 0 (negative: no positive signal), 1 (weak: light or dark red signal) and 2 (strong: bright red signal). Expression levels of Cav-1 were calculated based on the total score as the following equation: Intensity distribution (ID) = AP × IS.

The cutoff value for high and low expression was determined on the receiver operating characteristic (ROC) curve analysis with the respect to overall survival. According to the optimal sensitivity and specificity of the ROC curve by overall survival status, 1.5 was defined as the optimal cutoff for Cav-1 immunofluorescent score in CAFs (an ID score ≥1.5 defined high expression and ID score <1.5 indicated low expression), 3.5 was defined as the optimal cutoff for Cav-1 immunofluorescent score in tumor cells (an ID score ≥3.5 defined high expression and ID score <3.5 indicated low expression).

### Statistical Analysis

We used SPSS 17.0 software (Chicago, IL, USA) to carry out all statistical analyses. Friedman test was used to analyze the difference of Cav-1 original scores among different gastric lesions. ROC curve analysis was conducted to determine the cutoff point of high or low Cav-1 level and evaluate the predictive value of Cav-1 expression for overall survival. The χ2 test or Fisher’s exact test was used to analyze the correlation between the clinicopathological parameters and Cav-1 expression. Spearman’s rank correlation test was performed to analyze the association between Cav-1 expression in tumor cells and CAFs. The overall survival and disease free survival rate were estimated using the Kaplan-Meier method and compared using the long-rank test. Multivariate analysis using Cox proportional hazard regression model was used to test independent prognosis values. Two tailed *P*-values <0.05 were considered statistically significant.

## Results

### Expression of Cav-1 in Different Gastric Lesions

**Figure 1:**
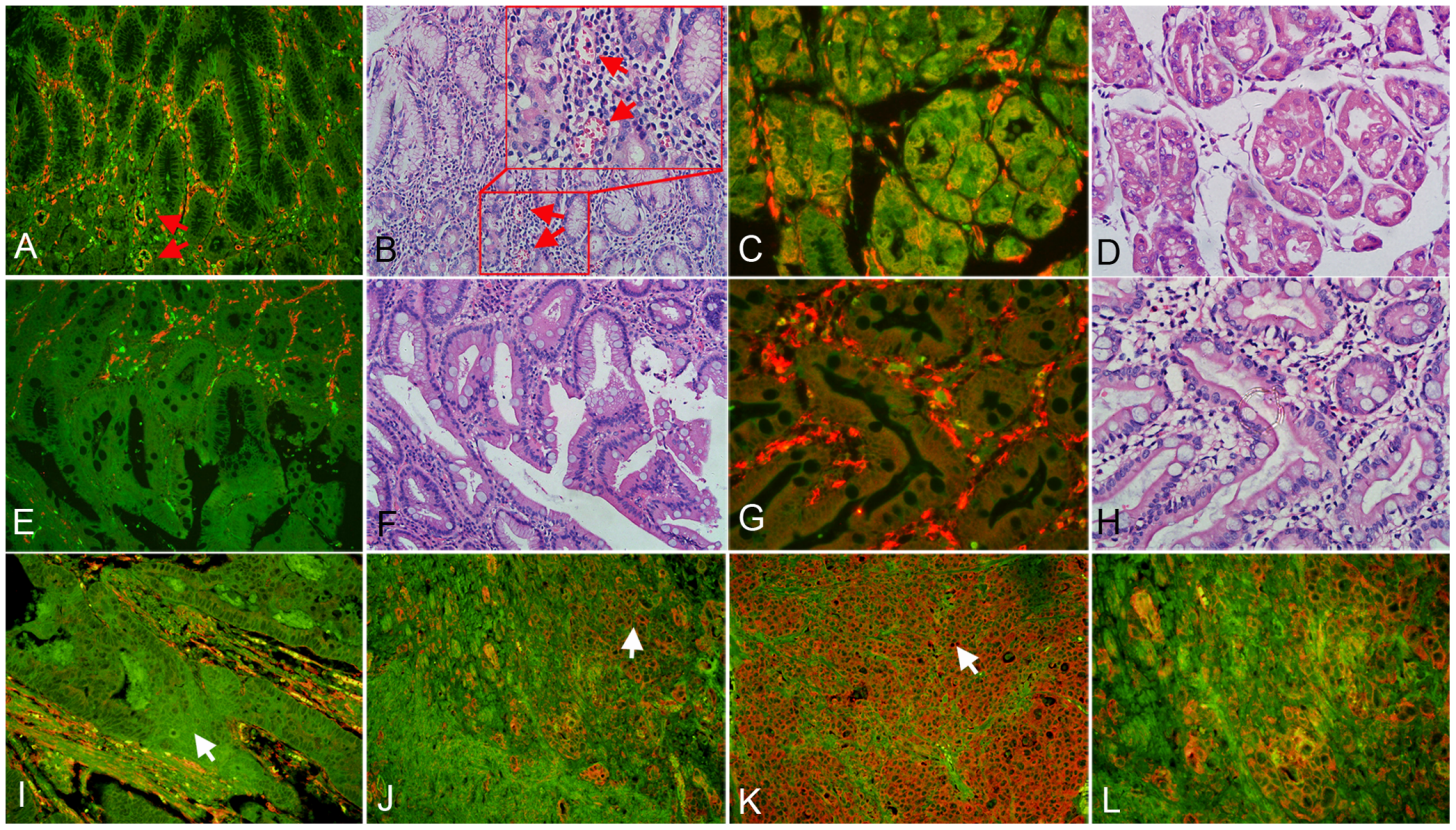
Cav-1 in gastritis with IM tissues/gastritis without IM tissues and signal calibration. A: Negative Cav-1 signal in gastritis without IM tissue, the red arrow indicates positive Cav-1 signal in blood vessels that used as internal positive control. **C:** Positive Cav-1 signal in gastritis without IM tissue. **E:** Negative Cav-1 signal in gastritis with IM tissue. **G:** Positive Cav-1 signal in gastritis with IM tissue. **I:** The white arrow shows negative signal (Intensity Score = 0). **J:** The white arrow shows weak signal (Intensity Score = 1). **K**: The white arrow shows strong signal (Intensity Score = 2). **L:** Cav-1 shows membrane-type staining. (**A, B, E, F, I, J, K:** 200× magnification; **C, D, G, H, L:** 400× magnification; **B, D, F** and **H** are H&E staining; **A** and **B**, **C** and **D**, **E** and **F, G** and **H** are serial sections, respectively).

**Figure 2:**
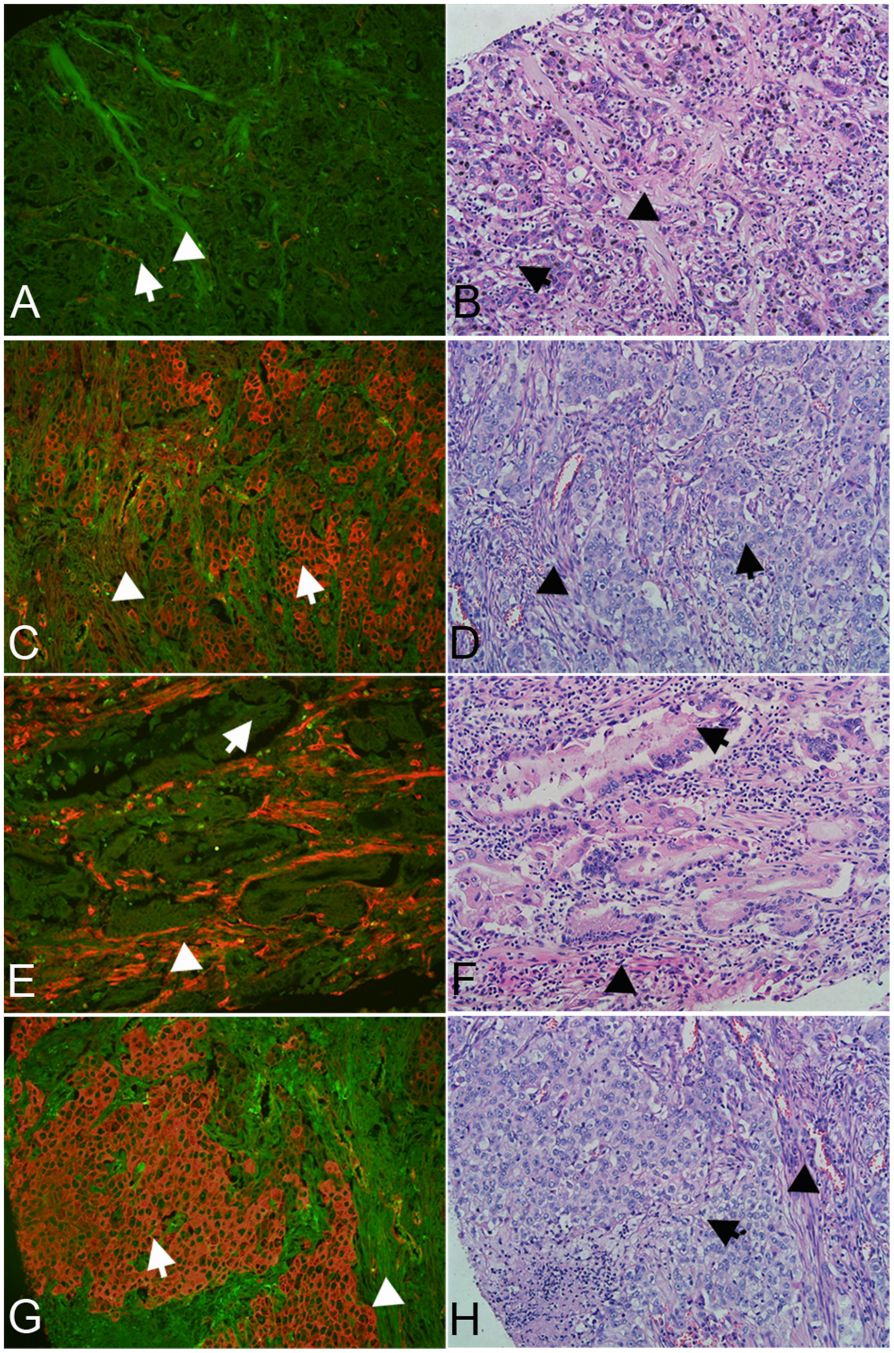
Representative examples of Cav-1 expression in GC. **A:** Negative Cav-1 expression in tumor cells and fibroblasts; **C:** Positive Cav-1 signal in tumor cells and fibroblasts; **E:** Positive Cav-1 signal was observed in fibroblasts, but negative in tumor cells; **G:** Positive Cav-1 signal in tumor cells, but negative in fibroblasts. (The white arrow shows the tumor cells and the white triangle shows the fibroblasts; **A-G:** 200× magnification; **B, D, F** and **H** are H&E staining; the parallel two pictures are serial sections respectively).

**Figure 3:**
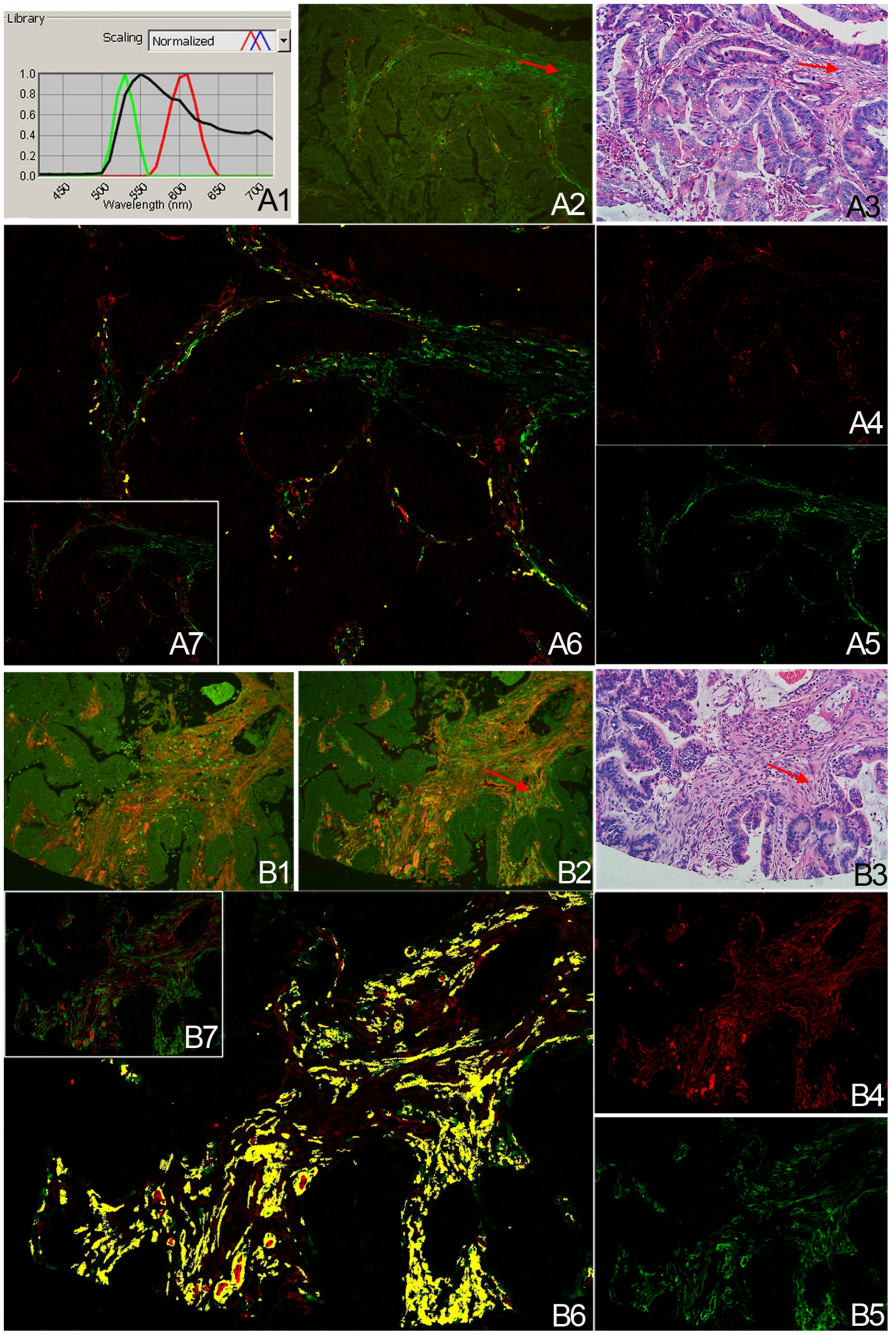
Co-localization of Cav-1 and α-SMA in GC tissue. **A1:** Emission spectra of QDs (525 nm-green; 605 nm-red) and tissue autofluorescence (black); Cav-1 signal is red and α-SMA is green. **A2–A7** shows few CAFs are Cav-1 positive and **B2–B7** shows most CAFs are Cav-1 positive. **A6, A7 and B6, B7:** co-localization of Cav-1 and α-SMA, the yellow pseudo-color in A6 and B6 indicates the co-localization of red and green signal; **A2, and B2:** the original images acquired by the multispectral microscopy imaging system; **A4, A5 and B4, B5:** the unmixed images. **A3 and B3:** H&E staining in serial sections. (The arrows show CAFs; B1 is single staining of Cav-1, we calibrated results of red signal between the single and double labeling to ensure comparability of different experiments, **A–B:** 200× magnification).

The localization and expression of Cav-1 in gastritis without IM, gastritis with IM and GC were assessed by QDs-IHC using the Cav-1 polyclonal antibody. The strong intensity doughnut-shaped positive signal of Cav-1 in the tumour stroma was the internal control of endothelial expression (**Fig. 1A**). In gastritis without IM tissues, negative Cav-1 signal predominantly in the surface mucous cells (**Fig. 1A**) and positive signal predominantly in the base and neck of fundic gland cells (**Fig. 1C**). In gastritis with IM tissues, some cases showed no Cav-1 distribution (**Fig. 1E**) and some showed positive Cav-1 staining in the absorptive cell and goblet cell of metaplastic glands (**Fig. 1G**). Expression of Cav-1 immunoreactivity was primarily observed at the cell membrane (**Fig. 1L**) and cytoplasm. The standard of signal scoring was displayed in Figure. 1I–K and representative examples of Cav-1 expression in GC were showed in **Figure. 2**. Colocalization of Cav-1 and α-SMA proteins was detected by QDs-based double immunofluorescent labeling in the GC, and analyzed by multispectral imaging systems, to precisely identify Cav-1 expression in CAFs (**Fig. 3**). The distribution of the signal of Cav-1 in fibroblasts is random and has no relation with the Cav-1 status in tumor cells.

Table 2 summarized the original scores of Cav-1 expression in gastritis without IM, gastritis with IM and GC. In epithelial compartment, 30% (6/20) of gastritis without IM, 55% (11/20) of gastritis with IM, 75% (15/20) of GC were scored 0,showing Cav-1 protein expression level decreasing gradually. Friedman test showed that the difference of Cav-1 staining score between gastritis without IM, gastritis with IM and GC was statistically significant (*P* = 0.012).

**Table 2:** Epithelial immunohistochemistry score distribution of different gastric lesions.

Original Score	Gastritis without intestinalmetaplasia n(%)	Gastritis with intestinal metaplasia n(%)	Gastric cancer n(%)
0	6(30.0)	11(55.0)	15(75.0)
1	0(0.0)	0(0.0)	0(0.0)
2	7(35.0)	6(30.0)	3(15.0)
3	6(30.0)	2(10.0)	1(5.0)
4	0(0.0)	0(0.0)	0(0.0)
6	1(5.0)	1(5.0)	0(0.0)
8	0(0.0)	0(0.0)	1(5.0)
Total	20	20	20
*P* value	**0.012**		

### Correlation Expression of Cav-1 between Tumor Cells and CAFs

Correlation between tumor cells and CAFs in Cav-1 expression was also analyzed. As was shown in Table 3, among 226 cases with low Cav-1expression in tumor cells, 121(53.5%) cases showed low expression of Cav-1 in CAFs (Fig. 2A), and among 21 cases with high Cav-1 expression in tumor cells, 9 (42.9%) cases displayed high Cav-1 expression in CAFs (Fig. 2C). The other 117cases showed distinct expression status in tumor cell and CAFs (Fig. 2E and G). No significant correlation was founded between tumor cells and CAFs Cav-1 expression (r = −0.20, *P* = 0.751).

**Table 3:** Correlations between Cav-1 expression and clinicopathological parameters of gastric cancer.

		Cav-1 in tumor cells				Cav-1 in CAFs		
Parameters	n	Low (%)	High (%)	*P*	n	Low (%)	HHigh (%)	*P*
**Age**				0.824				0.371
<57	131	122(93.1)	9(6.9)		112	64(57.1)	48(42.9)	
≥57	155	143(92.3)	12(7.7)		135	69(51.1)	66(48.9)	
**Gender**				0.618				0.157
Male	205	191(93.2)	14(6.8)		178	101(56.7)	77(43.3)	
Female	81	74(91.4)	7(8.6)		69	32(46.4)	37(53.6)	
**T stage^#^**				0.138				0.890
T1+T2	88	85(96.9)	3(3.1)		75	41(54.7)	34(45.3)	
T3+T4	198	180(90.9)	18 (9.1)		172	92(53.5)	80(46.5)	
**Lymph node status^#^**				0.937				0.321
N0	113	104(92.0)	9(8.0)		97	58(59.8)	39(60.2)	
N1	133	124(93.2)	9(6.8)		118	59(50.0)	59(50.0)	
N2	40	37(92.5)	3(7.5)		32	16(50.0)	16(50.0)	
**TNM stage^#^**				0.824				0.702
0+Ia+Ib+II	142	131(92.3)	11(7.7)		123	68(53.7)	55(46.3)	
IIIa+IIIb+IV	144	134(93.1)	10(6.9)		124	65(52.4)	59(47.6)	
**Grade of AC**				0.603				0.883
Well/Moderately	78	70(89.7)	8(10.3)		71	36(50.7)	35(49.3)	
Poorly	159	146(91.8)	13(8.2)		132	69(52.3)	63(47.7)	
**Lauren classification**				0.227				0.118
Intestinal-type	137	127(92.7)	10(7.3)		124	59(47.6)	65(52.4)	
Diffuse-type	123	116(94.3)	7(5.7)		101	62 (61.4)	39(38.6)	
Mixed-type	26	22(84.6)	4(15.4)		22	12(54.5)	10(45.5)	
**HER-2**				0.740				0.541
Negative	145	131(90.3)	14(9.7)		131	76(58.0)	55(42.0)	
Positive	34	32(94.1)	2(5.9)		30	15(50.0)	15(50.0)	
**Pathologic type**				0.096				0.095
AC	237	216(91.1)	21(8.9)		203	105(51.7)	98(48.3)	
Mucinous AC	32	32(100.0)	0(0.0)		29	21(72.4)	8(27.6)	
UC	17	17(100.0)	0(0.0)		15	7(46.7)	8(53.3)	
**Cav-1 in tumor cells**								0.751
Low (%)					226	121(53.5)	105(46.5)	
High (%)					21	12(57.1)	9(42.9)	

#TNM classification of malignant tumors (7th edition).

AC, Adenocarcinoma; UC, Undifferentiated carcinoma.

### Clinical Significance of Cav-1 Expression in Tumor Cells and CAFs of GC

All 286 GC samples were available for analysis of Cav-1 immunostaining in tumor cells and 247 (86.4%) cases had sufficient tissues for analysis of Cav-1 immunostaining in CAFs. Table 3 summarized the correlation between tumor cells or CAFs in Cav-1 expression and the classic clinicopathological parameters of GC, such as age, gender, T stage and lymph node status in univariate analysis. However, no significant relationship was found.

**Figure 4:**
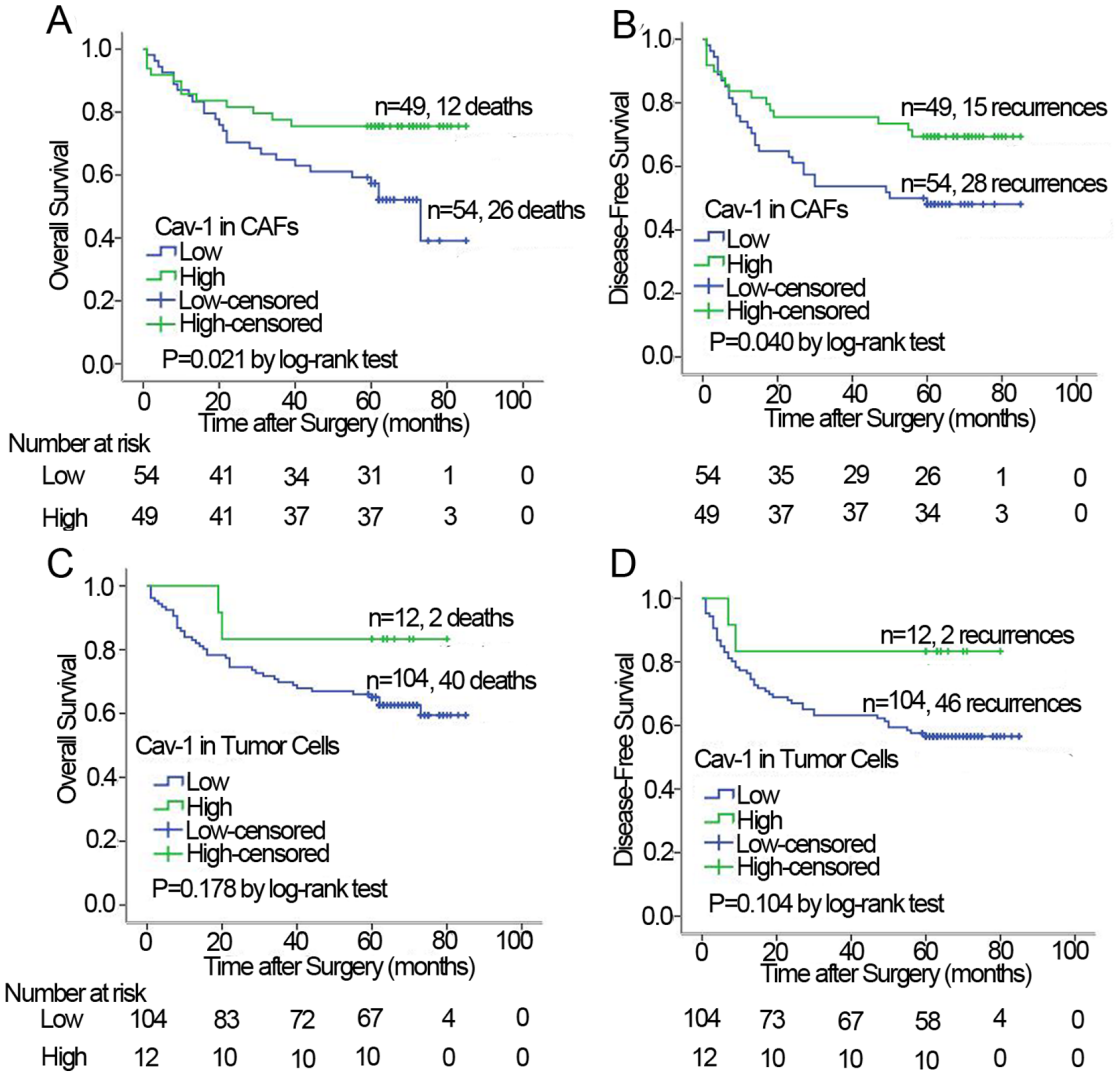
Cumulative survival curves of GC patients. **A and B:** The high level of Cav-1 in CAFs significantly correlates with overall survival and disease free survival. **C and D:** The high level of Cav-1 in tumor cells shows a better overall survival and disease free survival than the low, but it is not statistically significant.

Furthermore, we investigated the prognostic value of Cav-1 expression in tumor cells and CAFs. Kaplan-Meier analysis and the log-rank test showed that low expression of Cav-1 in CAFs rather than in tumor cells predicted poor survival. Median overall survival of low CAFs Cav-1 expression subgroup was 73.0 months (95% CI, 55.589–90.411), while in the follow-up interval, the high CAFs Cav-1 expression subgroup had a calculated survival rate of approximately 0.8 (**Fig. 4A**). As shown in Figure. 4B, low expression of Cav-1 in CAFs also predicted early recurrence of GC patients. Figure 4C and D indicated that tumor cells Cav-1 status had no significant correlation with overall survival and disease free survival (*P* = 0.178 and 0.104, respectively). Moreover, the cumulative 5 year survival rate of patients whose tumors exhibited high CAFs Cav-1 expression was 75.5% (95% CI, 0.642–0.868), whereas it was only 57.4% (95% CI, 0.456–0.692) in low CAFs Cav-1 group (*P* = 0.053).

In univariate analysis, TNM stage, T stage, lymph node status and CAFs Cav-1 level were found to be significantly associated with the disease free survival of GC patients (*P* = 0.001, 0.003, 0.010 and 0.029, respectively; Table 4). TNM stage, T stage and CAFs Cav-1 level were significantly correlated with the overall survival (*P* = 0.012, 0.006, and 0.013, respectively; Table 4). To determine whether CAFs Cav-1 expression was an independent predictor of GC patients’ recurrence and survival, a multivariate analysis was performed using COX proportional hazard regression model, together with age, T classification, TNM stage and other tumor features. Again, Cav-1 level in CAFs was independently and significantly associated with GC patients’ recurrence and outcome (*P* = 0.034 and 0.005, respectively; Table 4). On the contrary, tumor cells Cav-1 level failed to prognosticate GC patient’s recurrence and survival in the simultaneous model (Table 4).

**Table 4:** COX proportional hazard models on disease free survival and overall survival of GC patients.

	Univariate analysis		Multivariate analysis	
Factors	*P* value	HR(95%CI)	*P* value	HR(95%CI)
**Disease Free Survival**				
Sex (Male *vs*. Female)	0.868	0.959(0.589,1.561)		
Age (<57 *vs*.≥57)	0.156	0.725(0.462,1.140)	**0.019**	0.404(0.189,0.862)
TNM stage (I–II *vs*. III–IV)	**0.001**	0.419(0.238,0.736)	**0.005**	0.298(0.129,0.693)
T stage (T1–T2 *vs*. T3–T4)	**0.003**	0.384(0.181,0.815)		
Lymph node (No *vs*. Yes)	**0.010**	0.474(0.248,0.907)		
Lauren classification (Intestinal-type *vs*.Diffuse-type)	0.886	1.036(0.639,1.681)		
Grade of AC (Well and moderately *vs.* Poorly)	0.738	0.914(0.535,1.560)		
Cav-1 in CAFs (Low *vs*. High)	**0.029**	1.694(1.034,2.775)	**0.034**	2.188(1.063,4.504)
Cav-1 in tumor cells (Low *vs*. High)	0.074	2.604(0.721,9.400)		
**Overall Survival**				
Sex (Male *vs*. Female)	0.988	1.004(0.578,1.745)		
Age (<57 *vs*.≥57)	0.130	0.681(0.410,1.131)	**0.022**	0.390(0.175,0.871)
TNM stage (I–II *vs*. III–IV)	**0.012**	0.500(0.279,0.894)		
T stage (T1–T2 *vs*. T3–T4)	**0.006**	0.363(0.157,0.842)	**0.013**	0.215(0.064,0.720)
Lymph node (No *vs*. Yes)	0.061	0.558(0.288,1.081)		
Lauren classification (Intestinal-type *vs*.Diffuse-type)	0.759	0.923(0.552,1.543)		
Grade of AC (Well and moderately *vs.* Poorly)	0.886	0.965(0.595,1.565)		
Cav-1 in CAFs (Low *vs*. High)	**0.013**	1.966(1.118,3.456)	**0.005**	3.236(1.427,7.353)
Cav-1 in tumor cells (Low *vs*. High)	0.149	2.264(0.624,8.213)		

AC, Adenocarcinoma.

**Figure 5:**
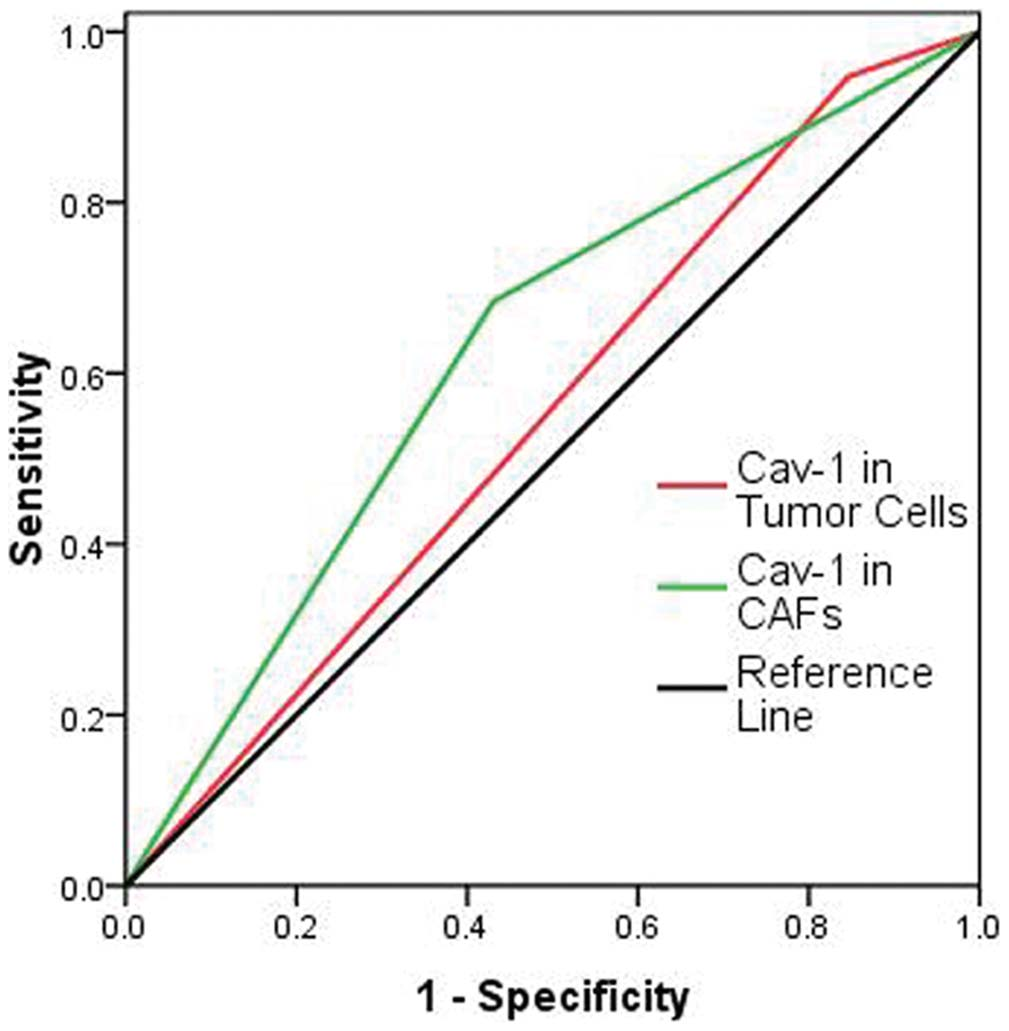
ROC analysis of Cav-1 scores by overall survival of GC patients. The cutoffs of tumor cell and CAFs Cav-1 have optimal sensitivity and specificity. Cav-1 expression level in CAFs (area under the curve was 0.627, 95% CI: 0.515–0.738; *P* = 0.032); Cav-1 in tumor cells (area under the curve was 0.551, 95% CI: 0.438–0.664; *P* = 0.393).

The death predictive value of Cav-1 thresholds in tumor cells and in CAFs was analyzed as well. As showed in Figure 5, Cav-1 level in CAFs predicted death with good performance; the area under the curve was 0.627 (95% CI: 0.515–0.738; *P* = 0.032). Whereas, Cav-1 level in tumor cells failed to predict death, with area under the curve of 0.551 (95% CI: 0.438–0.664; *P* = 0.393).

## Discussion

Here, we reported that Cav-1 immunoreactivity is mainly observed at the cell membrane and cytoplasm. In gastritis without IM tissues, Cav-1 signal is predominantly detected in the base and neck of fundic gland cells. In gastritis with IM tissues, Cav-1 staining mainly locates in the absorptive cell and goblet cell of metaplastic glands. This result correlates with another study which analyzed Cav-1 in gastric tissue via immunohistochemistry [27]. Cav-1 expression gradually decreases with the progression of GC and the expression level in CAFs rather than in tumor cells to a certain extent predicts recurrence and survival in GC patients.

Roles of Cav-1 protein in tumor development are tumor-dependent and compartment-dependent [12], [30]. Cav-1 gene is localized to locus D7S522 of human chromosome 7q31.1, which is often deleted in some types of tumors [11]. Moreover, Cav-1 is implicated in the caveolin scaffolding domain and can induce inhibition of cytokine receptor signaling [14], suggesting the tumor suppressor role of Cav-1. The tumor promoting activity of Cav-1 has been widely confirmed by carefully detecting its expression levels and clinical values in varieties of cancers, such as tongue squamous cell carcinoma, esophageal squamous cell carcinoma, bladder transitional cell carcinomas, nasopharyngeal carcinoma and neck squamous cell carcinoma [16], [31], [32], [33], [34]. Cav-1 has different roles in tumor cells and stromal cells. For example, in breast and prostate cancer, loss of stromal Cav-1 but not tumor cells Cav-1 heralds poor prognosis [6], [8], [12], [14], indicating the compartment-dependent roles of Cav-1.

Our results indicated that Cav-1 expression level in epithelia cells gradually decreased with the malignant progression of GC. Similar results were reported in another study [26]investigating Cav-1 expression of IM and GC, implying Cav-1 might be regarded as a tumor suppressor in the development of GC. Moreover, both tumor cells and CAFs Cav-1 protein expression status were not correlated to the typical clinicopathological parameters, such as T stage, TNM stage and Lauren classification. It was in contrast to the other study [26] which showed that the low expression of tumor cells Cav-1 protein was correlated with high T stage, TNM stage and lymph node metastasis. The difference might be due to the evaluation method: we evaluated the staining by multiplying the intensity and the proportion of cells stained and determined the cutoff point that had optimal sensitivity and specificity of the ROC curve based on overall survival status; whereas in the other research only the percentage of immunopositive cells was evaluated and the threshold of positive or negative was not presented. Besides, the larger sample size of our study influenced the results as well.

CAFs are among the most essential components of tumor stroma, with important functions inducing the deposition of ECM, regulating epithelial differentiation and inflammatory response and interacting with the microvasculature by secreting matrix metalloproteinases and vascular endothelial growth factor [35]. Since Cav-1 protein is the requisite structural component of caveolae, altered expression of Cav-1 protein in CAFs would impact various pathological processes and consequently promote tumor development. Loss of Cav-1 protein in CAFs promotes the activation of CAFs via activating TGF-β pathway, therefore facilitating the tumor microenvironment remodeling and tumor development [12]. However, another study published in *Cell* reported contrary functions of CAFs Cav-1 protein that stromal Cav-1 favors tumor invasion and metastasis through force-dependent architectural regulation of the microenvironment [36]. Therefore, the role of CAFs Cav-1 remains uncertain. The results presented here demonstrated an independent prognostic predictor of GC involving the Cav-1 expression level in CAFs. Low expression of Cav-1 in CAFs but not in tumor cells independently prognosticated early recurrence and poor survival of GC patients. ROC analysis indicated the death predicting value of low Cav-1 in CAFs. The results implied the tumor inhibiting role of CAFs Cav-1 protein and were consistent with the finding in breast and prostate cancer, which elucidated that loss of stromal Cav-1 heralds poor prognosis [8], [12], [15]. Also, in breast cancer, it is clear that with the development of cancer, the tumor microenvironment becomes a hypoxic and ill-nutrition niche, stimulating autophagy in CAFs. Then the abnormal activated autophagy degrades Cav-1 in CAFs. Of course, whether similar mechanisms exist in GC requires more *in vitro* studies.

Furthermore, loss of stromal Cav-1 has great predictive value in ER (+), PR (+), HER2 (+), and the so-called triple-negative breast cancer patients (ER(-)/PR(-)/HER2(-)).Concurrently instituted endocrine therapy does not influence its predictive value, making it a new universal or widely applicable breast cancer prognostic marker [37]. GC is the fourth common and third death causing cancer in the world [38], which still lacks enough appropriate biomarkers for prognostic evaluation and personalized anti-cancer treatment. The HER2 positive GC patients are benefited from trastuzumab treatment, however, only a few patients are HER2 positive, for example, only 19% of GC patients showed HER2 positive in our study. Nowadays, advanced GC patients with negative HER2 are still suffering from poor survival. Thus identifying more targets that suppress GC development is highly needed. Herein, we demonstrated the values of using stromal proteins as biomarkers in GC, which might contribute to the molecular typing of GC and consequently the treatment of GC, verifying the essential roles of tumor stroma in tumor development. Moreover, a prospective study is highly recommended to validate the prognostic value and to investigate the clinical significance of detecting Cav-1 in CAFs.

One interesting thing is that although CAFs Cav-1 status can be used as a predictor, when the correlation between CAFs Cav-1 levels and classic clinicopathological parameters of GC were analyzed, no significant association was found. Thus, promoting tumor invasion and metastasis may not be the primary mechanisms by which loss of CAFs Cav-1 promotes GC development. Chemotherapy is the most common adjunctive therapy that are recommended to the middle and advanced stage GC patients in China. Combined with the results shown here, we highlighted that whether low expression of Cav-1 in CAFs correlated with chemotherapy resistance may be a notable problem deserving further study.

Finally, in our study, via the advanced QDs-based double immunofluorescent labeling technology, Cav-1 protein in CAFs was precisely located. The pseudo-color co-localization images permitted us to evaluate the Cav-1 level in CAFs exclusively, however, QDs-IHC and QDs-based double immunofluorescent labeling technology is not commonly used in clinical laboratories. Immunohistochemistry is the most commonly practiced technology in clinical laboratories. Our previous study manifests that QDs-IHC method is as sensitive and specific as IHC [22]. If you want to detect simultaneously two different proteins such as Cav-1 and α-SMA in one slide, QDs-based double labeling is a novel, reliable and alternative method, by which we can get the results as precise as possible.

### Conclusions

In conclusion, our study evaluated the Cav-1 expression in both tumor cells and CAFs and investigated their clinical significance for GC patients. It is noteworthy that the expression of Cav-1 in CAFs rather than tumor cells particularly applicable in predicting early recurrence and poor survival prospects of GC patients, suggesting Cav-1 in CAFs may be a candidate therapeutic target and a useful prognostic marker of GC patients. Further studies are needed to investigate the mechanisms by which high levels of Cav-1 in CAFs suppress GC progression and, possibly, to develop therapeutics targeting Cav-1. Moreover, further clinical trials will be essential to verify the prognostic value of CAFs Cav-1 in GC and whether it could contribute to personalized anti-cancer treatment.
